# Association of Genetic Loci with Sleep Apnea in European Americans and African-Americans: The Candidate Gene Association Resource (CARe)

**DOI:** 10.1371/journal.pone.0048836

**Published:** 2012-11-14

**Authors:** Sanjay R. Patel, Robert Goodloe, Gourab De, Matthew Kowgier, Jia Weng, Sarah G. Buxbaum, Brian Cade, Tibor Fulop, Sina A. Gharib, Daniel J. Gottlieb, David Hillman, Emma K. Larkin, Diane S. Lauderdale, Li Li, Sutapa Mukherjee, Lyle Palmer, Phyllis Zee, Xiaofeng Zhu, Susan Redline

**Affiliations:** 1 Division of Sleep Medicine, Brigham and Women's Hospital, Boston, Massachusetts, United States of America; 2 Center for Human Genetics Research, Vanderbilt University Medical Center, Nashville, Tennessee, United States of America; 3 Department of Biostatistics, Harvard School of Public Health, Boston, Massachusetts, United States of America; 4 Samuel Lunenfeld Research Institute, Mount Sinai Hospital, Toronto, Ontario, Canada; 5 Jackson Heart Study, Jackson, Mississippi, United States of America; 6 Department of Epidemiology and Biostatistics, Jackson State University, Jackson, Mississippi, United States of America; 7 Department of Medicine, University of Mississippi Medical Center, Jackson, Mississippi, United States of America; 8 University of Washington Medicine Sleep Center and Center for Lung Biology, Division of Pulmonary and Critical Care Medicine, University of Washington, Seattle, Washington, United States of America; 9 Veterans Affairs Boston Healthcare System, Boston, Massachusetts, United States of America; 10 Framingham Heart Study, Framingham, Massachusetts, United States of America; 11 Department of Medicine, Boston University, Boston, Massachusetts, United States of America; 12 West Australian Sleep Disorders Research Institute, Sir Charles Gairdner Hospital, Perth, Australia; 13 Division of Allergy, Pulmonary and Critical Care, Department of Medicine, Vanderbilt University Medical Center, Nashville, Tennessee, United States of America; 14 Department of Health Studies, University of Chicago, Chicago, Illinois, United States of America; 15 Department of Family Medicine, Case Western Reserve University, Cleveland, Ohio, United States of America; 16 Department of Medicine, University of Toronto, Toronto, Ontario, Canada; 17 Women's College Research Institute, Women's College Hospital, Toronto, Ontario, Canada; 18 Department of Neurology, Northwestern University Feinberg School of Medicine, Chicago, Illinois, United States of America; 19 Department of Epidemiology and Biostatistics, School of Medicine, Case Western Reserve University, Cleveland, Ohio, United States of America; University of Jaén, Spain

## Abstract

Although obstructive sleep apnea (OSA) is known to have a strong familial basis, no genetic polymorphisms influencing apnea risk have been identified in cross-cohort analyses. We utilized the National Heart, Lung, and Blood Institute (NHLBI) Candidate Gene Association Resource (CARe) to identify sleep apnea susceptibility loci. Using a panel of 46,449 polymorphisms from roughly 2,100 candidate genes on a customized Illumina iSelect chip, we tested for association with the apnea hypopnea index (AHI) as well as moderate to severe OSA (AHI≥15) in 3,551 participants of the Cleveland Family Study and two cohorts participating in the Sleep Heart Health Study.

Among 647 African-Americans, rs11126184 in the pleckstrin (PLEK) gene was associated with OSA while rs7030789 in the lysophosphatidic acid receptor 1 (LPAR1) gene was associated with AHI using a chip-wide significance threshold of p-value<2×10^−6^. Among 2,904 individuals of European ancestry, rs1409986 in the prostaglandin E2 receptor (PTGER3) gene was significantly associated with OSA. Consistency of effects between rs7030789 and rs1409986 in LPAR1 and PTGER3 and apnea phenotypes were observed in independent clinic-based cohorts.

Novel genetic loci for apnea phenotypes were identified through the use of customized gene chips and meta-analyses of cohort data with replication in clinic-based samples. The identified SNPs all lie in genes associated with inflammation suggesting inflammation may play a role in OSA pathogenesis.

## Introduction

Obstructive sleep apnea (OSA) is a common disorder characterized by collapse of the upper airway during sleep leading to recurrent arousals, intermittent hypoxia, and surges in sympathetic activation. By narrowing the upper airway lumen, obesity is one of the strongest risk factors for OSA [1]. Independent of obesity, OSA has been implicated as an independent risk factor in the development of insulin resistance, hypertension and cardiovascular disease [2], [3], [4]. In addition, the fragmentation of sleep caused by OSA increases the risk of excessive daytime sleepiness and, as a result, risk of motor vehicle accidents [5], [6]. In total, OSA has been associated with substantial increases in healthcare spending [7].

Numerous studies have established that OSA aggregates within families suggesting the presence of a genetic predisposition [8], [9], [10]. Those with one affected relative are approximately 50% more likely to have OSA themselves [8]. Though obesity itself has a strong genetic basis, the familial aggregation of OSA persists even after accounting for obesity [11]. This may be due to the role of craniofacial morphology, ventilatory drive or other heritable traits important in OSA pathogenesis. The overall health impact of OSA and limited treatment options currently available for this disease underscore the need to better understand its molecular basis.

While prior genetic studies of OSA have suggested novel susceptibility loci, these studies have been relatively limited in terms of sample size. In this work, we sought to combine data from two of the largest sleep apnea epidemiologic cohorts, the Cleveland Family Study and the Sleep Heart Health Study, to identify genetic variants that predict OSA risk in both European ancestry and African-American populations.

These two cohorts participated in the NHLBI sponsored Candidate gene Association Resource (CARe) project wherein a custom candidate gene array assaying 45,237 single nucleotide polymorphisms (SNPs) over 2000 candidate regions relevant to heart, lung, blood, and sleep disorders [12], provided the ability to combine information from multiple cohorts in the search for genetic variants that influence risk for common diseases.

## Results

The CARe consortium included three cohorts with OSA phenotyping included in this analysis: the Cleveland Family Study (CFS) and subgroups of the Sleep Heart Health Study recruited from the Atherosclerosis Risk in Communities (ARIC) study and the Framingham Heart Study (FHS). In total, 3,551 individuals from these CARe cohorts were included in the primary analyses – 2,904 individuals of European ancestry (EAs) and 647 African-Americans (AAs). An additional 1795 individuals from the Western Australia Sleep Health Study (WASHS) served as a replication sample for the EA findings and 1010 cases and controls from the Cleveland Sleep Apnea (CSA) study and Case Transdisciplinary Research in Energetics and Cancer Colon Polyps Study (CTRECCPS) respectively served as a replication sample for AA findings. The sample size and participant characteristics from each study are shown in **Table 1**. There was a fairly even gender distribution in the community-based cohorts, whereas WASHS had a greater proportion of men as one would expect in a clinical cohort. In contrast, CSA and CTRECCPS had greater proportions of women likely reflecting gender differences in healthcare utilization among African-Americans [13]. Mean BMI across cohorts ranged from 28 to 41 kg/m^2^ suggesting a high prevalence of obesity. The prevalence of moderate to severe OSA was 29–36% across community-based cohorts.

**Table 1:** Characteristics of the Study Participants.

	European Ancestry				African-Americans		
Cohorts	ARIC (n = 1673)	FHS (n = 567)	CFS (n = 664)	WASHS (n = 1795)	CFS (n = 647)	CSA (n = 459)	CTRECCPS (n = 551)
Age (yrs)	62.5±5.7	59.2±9.0	41.3±19.6	51.5±13.2	38.6±19.2	49.1±14.4	56.0±8.8
Male	47%	48%	47%	63%	43%	33%	31%
BMI (kg/m^2^)	28.9±5.1	28.3±5.1	30.1±8.7	32.9±7.8	31.9±9.9	40.6±9.9	31.1±7.0
AHI (events/hr)	9.1 (3.7, 19.0)	8.0 (3.1, 17.7)	4.7 (1.4, 18.3)	28.5 (16.0, 50.7)	5.9 (1.5, 21.8)	39.2 (5.0, 192.5)	—
OSA	33%	29%	36%	77%	36%	100%	0%

Values displayed are means ± standard deviation or median (interquartile range) for continuous variables and percentages for dichotomous variables. OSA is defined as an AHI≥15 for ARIC, FHS, CFS, and WASHS, based on an AHI≥5 plus clinical symptoms for CSA, and based on physician diagnosis for CTRECCPS.

AHI: apnea hypopnea index; BMI: body mass index; OSA: obstructive sleep apnea.

Within the primary analytic sample, there was little evidence for population stratification in the EAs with genomic control inflation factors (λ) of 1.05 and 1.04 for the OSA and logAHI analyses respectively. Slightly greater λ values were obtained for AAs (1.10 for OSA and 1.08 for logAHI). However, after using genomic control correction, the resulting quantile-quantile plots (**Figures S1,S2,S3,S4**) showed no evidence of type I error inflation in any of the analyses.

### African-American Findings

**Figure 1:**
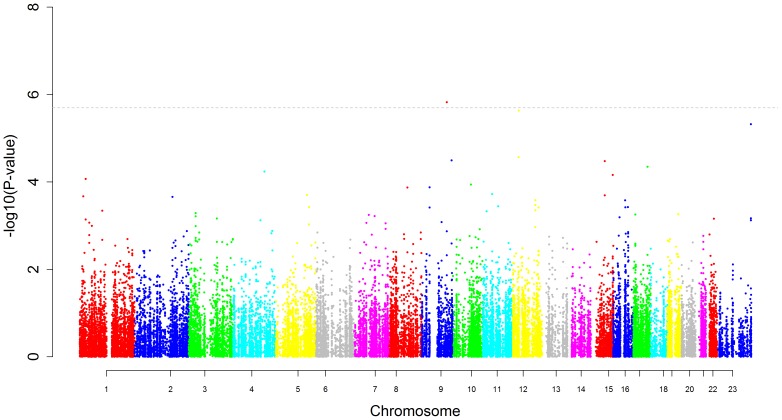
Manhattan plot for apnea hypopnea index in African-Americans. This figure plots the association results for all SNPs against log(apnea hypopnea index +1) among African-Americans. The y-axis displays the -log(p-value), the x-axis the SNP position on each chromosome. The dotted line represents the threshold for statistical significance based on the number of independent comparisons being made.

Manhattan plots displaying the strength of association between logAHI and OSA with each genotyped SNP in AAs are displayed in **Figures 1** **,** **2** respectively. One SNP, rs7030789 in an intronic region of the LPAR1 gene was significantly associated with logAHI in AAs (**Table 2**) while a second SNP, rs7972342, in an intronic region of the ITPR2 gene, just missed the threshold for statistical significance with a p-value of 2.3×10^−6^. The effects of these SNPs were reduced slightly in BMI-adjusted models compared to models not including BMI. However, stratified analyses revealed the effect of rs7030789 was actually greater in the non-obese than the obese (β = 0.18 vs. 0.09) and the effect of rs7972342 was similar in both groups (β = −0.12 vs. −0.15). Both SNPs were nominally associated with the dichotomous OSA phenotype (p = 0.007 for rs7030789 and p = 0.003 for rs7972342).

**Figure 2:**
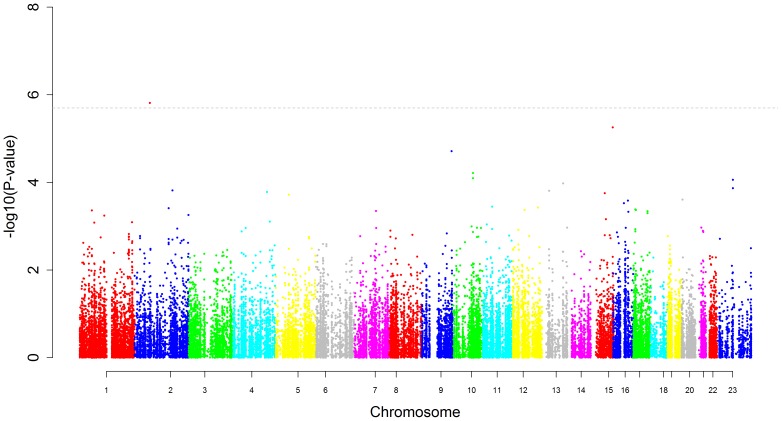
Manhattan plot for obstructive sleep apnea in African-Americans. This figure plots the association results for all SNPs against an apnea hypopnea index of 15 or greater among African-Americans. The y-axis displays the -log(p-value), the x-axis the SNP position on each chromosome. The dotted line represents the threshold for statistical significance based on the number of independent comparisons being made.

**Table 2:** SNPs associated with AHI.

									BMI-Adjusted		BMI-Unadjusted	
Ethnicity	SNP	Gene	Chr	Position	Minor Allele	Major Allele	Minor Allele Frequency	Hardy-Weinberg P-value	Beta (SE)	P-value	Beta (SE)	P-value
African-American	rs7030789	LPAR1	9	112775333	A	G	0.319	0.10	0.109 (0.023)	1.50×10^−6^	0.129 (0.028)	4.58×10^−6^
African-American	rs7972342	ITPR2	12	26681785	A	G	0.290	1.00	−0.113 (0.024)	2.33×10^−6^	−0.134 (0.030)	6.97×10^−6^

One SNP, rs11126184 downstream of the PLEK gene on chromosome 2 was significantly associated with the dichotomous OSA phenotype (**Table 3**). The minor allele in this SNP was associated with a reduced risk of OSA with an OR of 0.43. The protective effect of this SNP was not reduced with BMI adjustment and in fact, stratified analyses suggested a stronger association in the non-obese (OR = 0.26) compared to the obese (OR = 0.47) subgroup. This suggests rs11126184 may influence OSA through obesity-independent pathways. This SNP was nominally associated with logAHI (p = 0.004).

**Table 3:** SNPs associated with OSA.

										BMI-Adjusted		BMI-Unadjusted	
Ethnicity	SNP	Gene	Chr	Position	Minor Allele	Major Allele	Minor Allele Frequency	I^2^	P-value*	OR (95% CI)	P-value	OR (95% CI)	P-value
African-American	rs11126184	PLEK	2	68505678	A	C	0.378	—	0.36	0.43 (0.31, 0.60)	1.54×10^−6^	0.45 (0.33, 0.62)	1.41×10^−6^
European Ancestry	rs1409986	PTGER3	1	71104086	A	G	0.075	32%	0.23	2.14 (1.58, 2.90)	1.01×10^−6^	1.77 (1.30, 2.41)	2.21×10^−4^

* Heterozygosity P-value for European ancestry cohorts and Hardy-Weinberg P-value for African-Americans.

### African-American Replication

Of the three SNPs in AAs with evidence of association, genotyping of rs7972342 did not meet quality control standards. The other two SNPs were tested for association with the OSA phenotype. No evidence of deviation from HWE was found for either SNP among controls. No evidence for association was found with rs11126184 (OR = 1.03, p = 0.40). In contrast, an association was noted between rs7030789 and OSA status with an OR of 1.29 (p = 0.01) for each additional risk allele after adjusting for age, sex, and BMI.

### European Ancestry Findings

**Figure 3:**
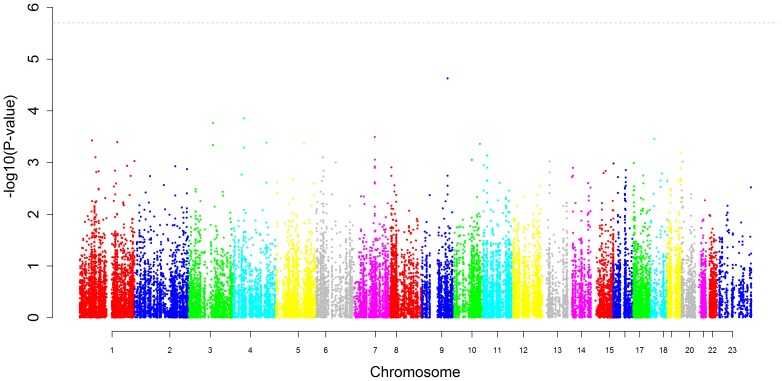
Manhattan plot for apnea hypopnea index in the European ancestry population. This figure plots the association results for all SNPs against log(apnea hypopnea index +1) among those of European ancestry. The y-axis displays the -log(p-value), the x-axis the SNP position on each chromosome. The dotted line represents the threshold for statistical significance based on the number of independent comparisons being made.

Manhattan plots displaying the strength of association between logAHI and OSA with each genotyped SNP in EAs are displayed in **Figures 3** **,** **4** respectively. Among EAs, no SNP met criteria for a statistically significant association with logAHI. One SNP, rs1409986, located in an intronic region of the PTGER3 gene which codes for a prostaglandin E2 receptor was significantly associated with OSA in EAs (p = 1.0×10^−6^), with an OR of 2.1 for each additional risk allele (**Table 3**). This SNP did not meet quality standards in the ARIC cohort. However, between the other two cohorts, a formal test for heterogeneity (I^2^ = 0.32, p = 0.22) was nonsignificant with an OR = 1.82 in CFS and an OR = 2.78 in FHS. The association strengthened with adjustment for BMI suggesting that this association was not mediated through obesity. In support of this interpretation, analyses stratified by obesity status found similar effects for rs1409986 among those with and without obesity (OR = 2.36 vs. 2.37). This SNP was nominally associated with logAHI (p = 0.02).

**Figure 4:**
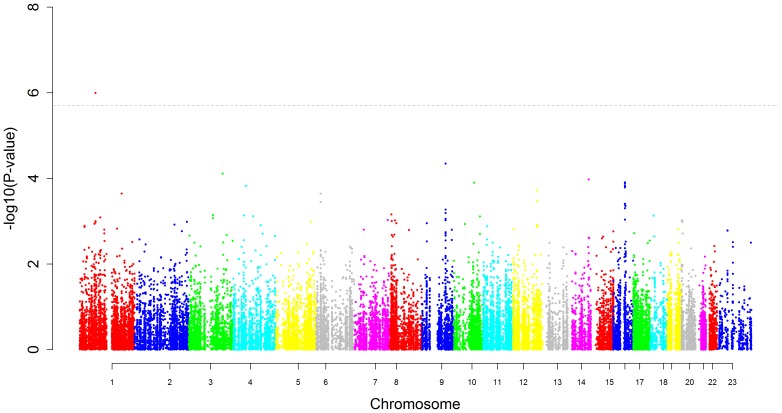
Manhattan plot for obstructive sleep apnea in the European ancestry population. This figure plots the association results for all SNPs against an apnea hypopnea index of 15 or greater among those of European ancestry. The y-axis displays the -log(p-value), the x-axis the SNP position on each chromosome. The dotted line represents the threshold for statistical significance based on the number of independent comparisons being made.

### European Ancestry Replication

The minor allele frequency for rs1409986 in WASHS was 7.3% with no evidence of deviation from HWE (p = 0.38). The OR for OSA for each risk allele in this cohort adjusting for age, sex, and BMI was 1.20 (p = 0.13). Under an additive model, there was no association with logAHI (p = 0.35). However, under a co-dominant model, an association was found (p = 0.05). While no difference was found between CT and CC genotypes, the TT genotype was associated with a 50% greater AHI than the wild type CC genotype (p = 0.02).

### Sensitivity Analyses

No change in results for either EAs or AAs was found by including data from the 354 children in the CFS cohort. In contrast, results from a third Sleep Heart Health Study cohort, the Cardiovascular Health Study (CHS), did not support findings for either ethnic group. Note that this cohort was on average more than 10 years older than both ARIC and FHS with an even greater age difference with CFS.

Comparing results across ethnic groups, the prevalence of the minor allele for rs1409986 (PTGER3) in AAs was only 2%, precluding accurate estimation of its effect in this population. Among the top three SNPs for apnea-related traits identified in AAs, rs7030789 (LPAR1) showed evidence for association to an apnea phenotype in EAs with p = 0.01 for association to OSA and p = 0.06 to logAHI.

## Discussion

This study represents one of the first systematic evaluations of a large number of genetic loci identified as relevant to heart, lung, blood, and sleep phenotypes in relationship to sleep apnea. OSA represents one of the most common sleep disorders with a prevalence of 2 to 4% in children and greater than 10% in adults [14], [15]. The substantial neurocognitive and cardiovascular morbidity attributed to OSA as well as the likely rising prevalence of this condition with the growing obesity epidemic make understanding the genetic basis for this disease an important priority. Using genetic studies to identify novel molecular pathways involved in OSA pathogenesis may allow for the development of new treatments for this disorder – an important goal given the relatively poor tolerance of current treatment options.

Using data collected from three large cohorts and a genotyping platform that selected for candidate genes relevant to cardiovascular, respiratory and sleep physiology, we identified several SNPs associated with OSA related phenotypes.

Among AAs, the lysophosphatidic acid receptor 1 (LPAR1) gene was identified as a potential susceptibility locus. The rs7030789 SNP in an intronic region of LPAR1 was significantly associated with AHI in the discovery sample, demonstrated nominal association with the OSA phenotype in the CARe dataset as well as in the replication analysis comparing clinical OSA cases to controls. In addition, this locus showed evidence for replication in EAs strengthening the evidence base for a true susceptibility locus near this SNP. A locus close to LPAR1 has been shown to increase LPAR1 expression and subsequently increase circulating monocyte numbers [16], [17], suggesting a pro-inflammatory role for this gene. In addition, LPAR1 is expressed in proliferating regions of the embryonic cerebral cortex [18]. An LPAR1 knockout mouse model has been found to have abnormal innate behaviors as well as craniofacial abnormalities [19], suggesting potential neural and/or skeletal mechanisms for OSA pathogenesis.

The other loci identified in the CARe cohorts included one in plekstrin (PLEK), which is a substrate for protein kinase C in platelets and a wide range of leukocytes including monocytes and macrophages. This gene plays a role in actin assembly; knock out of PLEK in mouse models results in defective exocytosis [20]. In addition, there was suggestive evidence for an association with a locus in the gene for the inositol triphosphate receptor 2 (ITPR2). The ITPR2 gene plays a central role in intra-cellular calcium regulation, which is vital to cellular stability, cellular adhesion, and second messenger activity. Prior candidate gene studies have associated SNPs in ITPR2 with markers of inflammation and endothelial dysfunction as well as blood pressure phenotypes [21], [22]. Together with the LPAR1 findings, these results suggest alterations in inflammatory pathways including monocyte/macrophage function may be particularly important in OSA pathogenesis among AAs as well as the possibility of overlapping pathways that may mediate both hypertension and OSA. However, this connection between loci associated with inflammation and OSA cannot distinguish between the possibilities that inflammation is a primary causal factor for OSA versus that inflammation plays a secondary role in increasing OSA severity among those with an underlying predisposition.

In EAs, a polymorphism in PTGER3, a prostaglandin E2 receptor, was significantly associated with OSA. In a replication analysis using data from a clinic-based cohort which had a skewed distribution of AHI due to high representation of patients with sleep apnea or sleep apnea symptoms, this SNP was associated with an incremental increase in disease severity as measured by the AHI level. Although association with the dichotomous trait OSA was not replicated, this may be due to limited power for this trait since nearly 80% of subjects in the WASHS cohort met OSA criteria. The PTGER3 gene is expressed in neuronal tissue and modulates neurotransmitter release in both central and peripheral neurons. A haplotype analysis of PTGER3 recently suggested this gene may represent a risk factor for hypertension [23]. Unfortunately, sleep apnea was not characterized in that study.

It is important to note that our findings were not replicated in the Cardiovascular Health Study. This cohort is substantially older than the cohorts from which the primary results are based and the mechanisms underlying OSA in the elderly may differ substantially from OSA in middle aged populations. For example, obesity plays a much weaker role in the elderly while issues related to ventilatory control are much more prominent as evidenced by the greater number of central events in older individuals. Only one of the associations (rs7030789 in LPAR1) could be replicated across both EAs and AAs. This may be due to differences in allele frequencies impacting power (as for rs1409986 in PTGER3), differences in linkage disequilibrium structure or other ancestry related effects.

A prior analysis of CFS data has also utilized a candidate gene approach [24]. In that study, 53 candidate genes for OSA were genotyped and three loci were identified meeting chip-wide significance criteria, 2 in EAs (loci in C-reactive protein - CRP and glial cell derived neurotrophic factor - GDNF) and 1 in AAs (locus in serotonin 2A receptor - HTR2A). None of the top loci from this study were included as candidate genes in the prior report and one of the top loci from that report (GDNF) was not genotyped on the IBC platform used here. For the two overlapping loci (CRP and HTR2A), associations with apnea phenotypes were again confirmed among CFS EAs and AAs, although the level of statistical significance did not meet the stricter threshold used in this paper due to the greater number of SNPs interrogated. Of note, the CRP locus did not replicate among EAs from ARIC and FHS, demonstrating the importance of replication cohorts.

Of interest, although the African American sample was relatively small, it was derived from a single cohort that was assembled explicitly to enhance the power to detect genetic associations through recruitment of affected probands and multiplex families. OSA is common in minority populations and prior segregation analyses found stronger transmission of one or several alleles in AAs compared to EA families [25]. Although genetic studies in AAs may be limited by the coverage patterns of existing chips, the genetic diversity of this admixed population also provides opportunities to identify novel variants and to perform in silico “fine mapping” [26]. Despite the sample size, we identified two loci significantly associated with sleep apnea phenotypes in AAs and a third nearly significant association. Furthermore, we were able to replicate the strongest finding in an independent sample.

Strengths of this study include its use of standardization in both phenotyping and genotyping across several cohorts. For the primary analyses, sleep apnea was objectively measured from overnight polysomnography by a central laboratory minimizing measurement error and all genotyping was conducted by a single laboratory. The use of a broadly defined candidate gene approach (i.e., with genes selected for heart, lung, blood and sleep traits) provides balance in examining relevant but not narrowly defined susceptibility loci without the statistical penalties incurred by using a genome-wide association approach. This is particularly relevant for conditions such as OSA that are expensive to phenotype. On the other hand, this approach is not amenable to identifying completely novel loci.

We also conducted analyses in parallel to identify loci important in both African-Americans and those of European ancestry. Demonstrating a similar pattern of association between apnea phenotypes and rs7030789 in both groups provides additional evidence as to the presence of a true association. Another strength of the current study is the use of both principal component adjustment and genomic control correction to prevent inflation of the false positive rate due to cryptic population stratification or other sources of bias. Nevertheless, additional analyses from independent cohorts are needed to replicate the findings presented in this study.

Limitations of the work include the relatively small number of cohorts included and modest sample size compared to other cross-cohort genetic analyses. Unfortunately, few cohorts with detailed sleep apnea phenotyping as well as genotyping currently exist, particularly in minority populations. This limited our opportunity to conduct replication studies which will be needed to definitively confirm our findings. Furthermore, it should be noted that no functional data are yet available to demonstrate a direct effect of any of the variants identified with pathophysiological changes relevant to OSA.

Overall, the relatively small number of loci identified and replicated in this study presumably reflects a combination of the limited power to detect variants with low prevalence or small effect sizes and that many of the genetic variants contributing to OSA risk are either rare, have fairly modest effects, or both. This is in line with current views that OSA represents a complex disease having many genetic and environmental contributors each of which explains a relatively small proportion of the total attributable risk. The limited success in identifying genetic determinants of OSA might also reflect heterogeneity in genetic etiology across the various cohorts studied which differ in mean age and BMI. In fact, the association between OSA and rs7030789 appeared to be much stronger among leaner individuals. Another possible explanation is that causal variants with relatively large effects may exist in regions of the genome that were not interrogated in this candidate gene approach. Genome wide association (GWA) study designs will be required to assess whether such variants exist.

In summary, our results identify several loci, each of which has been implicated in inflammation, associated with a sleep apnea phenotype. While much work has focused on the potential pro-inflammatory effect of OSA, inflammation may play a causal role in OSA pathogenesis as well. Inflammation is prominent in both the mucosal and muscular layers of upper airway tissues of patients with OSA [27], [28]. In addition, inflammation may also impact ventilatory or sleep-wake control mechanisms relevant to OSA pathogenesis.

Of note, though all of the cohorts studied had a high prevalence of obesity, the identified associations were independent of obesity supporting the importance of identifying pathways leading to OSA within an obese population. Additional studies are needed to better understand the molecular pathways that increase susceptibility to this highly prevalent disorder, the overlap of such pathways with ones that also mediate cardiopulmonary disorders, and the extent to which the associations are moderated by ancestral background.

## Materials and Methods

### Study Sample

The CARe Consortium is a NHLBI supported resource for analyses of the association of genotypes with heart, lung, blood, and sleep phenotypes. CARe is composed of 9 large cohort studies of which four have collected polysomnographic data in at least a subset. These are the Cleveland Family Study (CFS), and subsets of three cohorts – the Atherosclerosis Risk in Communities (ARIC) study, the Cardiovascular Health Study (CHS), and the Framingham Heart Study (FHS) – which participated in the Sleep Heart Health Study (SHHS). Because the OSA phenotype changes across the lifespan with differing risk factors in pediatric, middle-aged, and elderly populations, we focused on the middle-aged. Thus, all children (age<18 yrs) from the CFS cohort and the entire CHS cohort (with mean age at the time of OSA phenotyping of 72 yrs) were excluded from the primary analyses.

CFS is a family-based longitudinal cohort study designed to study the genetic basis of OSA [8]. Index probands with a laboratory confirmed diagnosis of OSA, and at least two first-degree relatives available to be studied were recruited along with family members. Initially, neighborhood controls and their relatives were also recruited. Overall, a total of 2284 individuals from 361 families were recruited from the Cleveland metropolitan area. DNA was available in a subset of 1665 individuals for CARe genotyping. Phenotype data was used from the last available exam.

SHHS is a prospective cohort of 6,441 subjects who underwent polysomnography recruited from six established cohorts to study the effects of OSA on cardiovascular disease [29]. Three of the six parent cohorts contributing to SHHS, ARIC, FHS, and CHS, participated in CARe and so contributed genetic material. SHHS recruited subjects over the age of 40 with oversampling of those reporting a history of snoring at some sites. Full details have been previously published [29].

ARIC is a longitudinal cohort study of atherosclerosis and its clinical sequelae. From 1987 to 1989, a population-based sample of 15,792 men and women aged 45 to 64 years were recruited from 4 US communities (Forsyth County NC, Jackson MS, suburban Minneapolis MN, and Washington County MD). SHHS recruited 1000 ARIC participants in Minneapolis and 750 participants from Washington County, with oversampling of habitual snorers.

The FHS started in 1948 with 5209 randomly ascertained adult participants from Framingham, MA. In 1971, the Offspring cohort (comprised of 5124 individuals who were children of the original cohort participants or the children's spouses) was created. SHHS recruited 699 FHS participants from the Offspring cohort, all of European ancestry.

The Western Australia Sleep Health Study (WASHS) is a prospective cohort study of patients referred to the sole public sleep clinic in Western Australia. It has been designed to identify the genetic basis of sleep disorders and co-morbidities [30]. The vast majority (91%) of participants were referred for sleep apnea. Recruitment began in 2006. The cohort is predominantly of European ancestry. Analyses are limited to the first 1795 individuals of European ancestry with both DNA and sleep phenotyping available.

The Case Sleep Apnea (CSA) cohort recruited patients referred for evaluation of obstructive sleep apnea in the University Hospitals Case Medical Center sleep disorders clinic and laboratory from 2007 to 2010. Of 945 patients diagnosed with OSA based on symptoms and an AHI>5, 475 reported African-American background. Of these genotyping was successfully performed on 459.

These cases were matched against controls derived from the Case Transdisciplinary Research in Energetics and Cancer Colon Polyps Study (CTRECCPS) cohort. Description of this cohort has been previously reported [31]. Individuals over age 30 referred for routine screening colonoscopy at an endoscopy center affiliated with University Hospitals Case Medical Center were recruited providing a study base similar to that for the CSA cohort. Of 1259 subjects recruited from 2006 to 2009, 600 reported African-American background. Forty-nine individuals reporting a clinical diagnosis of sleep apnea were excluded from this analysis leaving a control sample size of 551.

The protocols for creating each of the cohorts and data collection were approved by the Institutional Review Board or Ethics Committee at Partners Health Systems (CARe), Johns Hopkins University (ARIC), University of Minnesota (ARIC), University Hospitals Case Medical Center (CFS, CSA, CTRECCPS), Boston University Medical Center (FHS), and Sir Charles Gairdner Hospital (WASHS) and all participants provided written informed consent.

### Sleep Apnea Phenotyping

Quantification of sleep apnea severity was done using polysomnography as part of either the SHHS or the CFS, using techniques that have been previously described [32], [33]. In brief, SHHS participants underwent in-home polysomnography using the Compumedics P Series System (Abbotsville, Australia) while CFS participants underwent in laboratory polysomnography using the Compumedics E series system or in-home sleep studies with measurement of oximetry, effort, thermistry, and body position (Edentec, Eden Prairie, MN). Indices derived from either technique were highly correlated [34]. Scoring of both sleep and breathing was done in a standardized fashion across SHHS and CFS by the same Reading Center at Case Western Reserve University [32]. Apneas were defined as no airflow for 10 seconds while hypopneas were defined as a 30% reduction in airflow accompanied by a 3% desaturation or arousal. Overnight in-laboratory polysomnography was performed in WASHS and the Case Sleep Apnea cohort and the AHI was computed using similar scoring criteria as recommended by the American Academy of Sleep Medicine [35]. Two phenotypes were assessed for association with candidate SNPs. A quantitative trait, apnea hypopnea index, was log-transformed (after adding 1) to approximate a normal distribution. In addition, the dichotomous trait OSA, defined as an AHI≥15, was assessed.

### Covariate Assessment

Age, gender, and ethnic background were obtained by self-report. Height and weight were measured in a standardized fashion in each cohort and body mass index (BMI) calculated as the ratio of weight to height squared.

### Genotyping

Genotyping was performed at the Broad Institute utilizing an IBC 50K SNP array which was custom designed as a gene-centric single nucleotide polymorphism (SNP) genotyping array that contains greater SNP marker density and linkage disequilibrium coverage for over 2000 candidate regions relevant to heart, lung, blood and sleep disorders than current genome-wide arrays, including approximately 7800 SNPs not present in the HapMap. Candidate loci were identified based on a literature search as well as pathway-based tools for relevant biologic pathways, and unpublished cardiovascular expression quantitative trait loci (eQTL) and GWA data. Loci were selected for coverage based on a voting process by consortium investigators to rank loci by importance. Further details have been previously published [12]. A total of 45,237 candidate SNPs were genotyped on the IBC array. The tagging approach utilized on the IBC array was designed to capture maximal genetic information from the HapMap populations as well as European and African American individuals from the SeattleSNPs and NIEHS sequencing programs [12]. Only SNPs with a MAF of 5% or greater were included in these analyses due to concerns for unstable point estimates and inflated type 1 error. SNPs were clustered into genotypes using the Illumina Beadstudio software and subjected to quality control filters at the sample and SNP level, separately within each cohort. Samples were excluded for individual call rates <90%, gender mismatch and duplicate discordance. SNPs were removed for call rates <95% or Hardy-Weinberg equilibrium (HWE) p<10^−5^ in analyses of individuals of European ancestry (EAs). Because of expected admixture, no HWE filtering was used for African-Americans (AAs).

### Statistical Analysis

#### Principal Components Analysis (PCA)

Principal components were generated using EIGENSTRAT within each cohort using the CARe genotype data in order to control for population stratification [36], [37]. Two reference populations were included in the principal component analysis of AAs: 1,178 European Americans from a multiple sclerosis GWA study (from Dr. Phil de Jager and colleagues), and 756 Nigerians from the Yoruba region from a hypertension GWA (provided by Dr. Richard Cooper and colleagues). Importantly, these two samples underwent extensive quality control procedures to remove population outliers using PCA. Ten principal components were generated for each cohort.

#### Within Cohort Analysis

All data analyses were performed separately for EAs and AAs. To further adjust for population stratification, the first 10 principal components were incorporated as covariates in all analyses. Primary analyses adjusted for BMI to identify variants whose actions were independent of obesity. Secondary analyses assessed the strength of association without BMI adjustment.

Analyses were carried out using a linear or logistic statistical framework for logAHI and OSA respectively in PLINK V 1.0.7 [38], or, for cohorts that included related individuals (CFS and FHS), R scripts that model family structure were used [39]. Consistency of findings was evaluated by comparing results in secondary analyses stratified on obesity status as defined by a BMI≥30 kg/m^2^.

#### Meta-analysis

For EAs, results from each of the three cohorts (CFS, ARIC, FHS) were combined using a fixed effects meta-analysis with an inverse-variance weighted approach as implemented in METAL [40]. Heterogeneity was assessed using the *I* ^2^ inconsistency metric. No meta-analysis was required for AAs as only CFS had sufficient AAs for analysis.

#### Genomic Control

Genomic control correction was applied after calculating the genomic control inflation factor (λ) using METAL based on the results of the meta-analysis performed in EAs or in the CFS cohort alone for AAs.

#### Significance Criterion

To correct for multiple testing, we determined the effective number of independent tests as 26,482 for AA and 20,544 for EA based on the correlation structure among the SNPs estimated using the spectral method [41], [42]. To maintain an overall type 1 error rate of 5%, a statistical threshold of α = 2×10^−6^ was used to declare array-wide significance.

#### Replication Analyses

Replication of EA findings was assessed using the WASHS cohort while replication of AA findings used case control data with cases from the CSA cohort and controls from the CTRECCPS cohort. Statistical analyses were done using linear and logistic regression for logAHI and OSA respectively adjusting for age, sex, and BMI and one-sided p-values were computed.

## Supporting Information

Figure S1 Quantile-quantile plot for apnea hypopnea index in African-Americans. This figure plots expected versus observed p-values from the association analyses of all SNPs against log(apnea hypopnea index+1) in African-Americans. The plotted observed p-values are after accounting for genomic control.
(TIF)

Figure S2 Quantile-quantile plot for obstructive sleep apnea in African-Americans. This figure plots expected versus observed p-values from the association analyses of all SNPs against an apnea hypopnea index of 15 or greater in African-Americans. The plotted observed p-values are after accounting for genomic control.
(TIF)

Figure S3 Quantile-quantile plot for apnea hypopnea index in European ancestry individuals. This figure plots expected versus observed p-values from the association analyses of all SNPs against log(apnea hypopnea index+1) in those of European ancestry. The plotted observed p-values are those from the meta-analysis after accounting for genomic control.
(TIF)

Figure S4 Quantile-quantile plot for obstructive sleep apnea in European ancestry individuals. This figure plots expected versus observed p-values from the association analyses of all SNPs against an apnea hypopnea index of 15 or greater in those of European ancestry. The plotted observed p-values are those from the meta-analysis after accounting for genomic control.
(TIF)
