# Supplementary figures and images for: Association of Genetic Loci with Sleep Apnea in European Americans and African-Americans: The Candidate Gene Association Resource (CARe)
Source: PLoS One. 2012 Nov 14;7(11):e48836. doi: 10.1371/journal.pone.0048836 (PMC3498243; doi:10.1371/journal.pone.0048836)

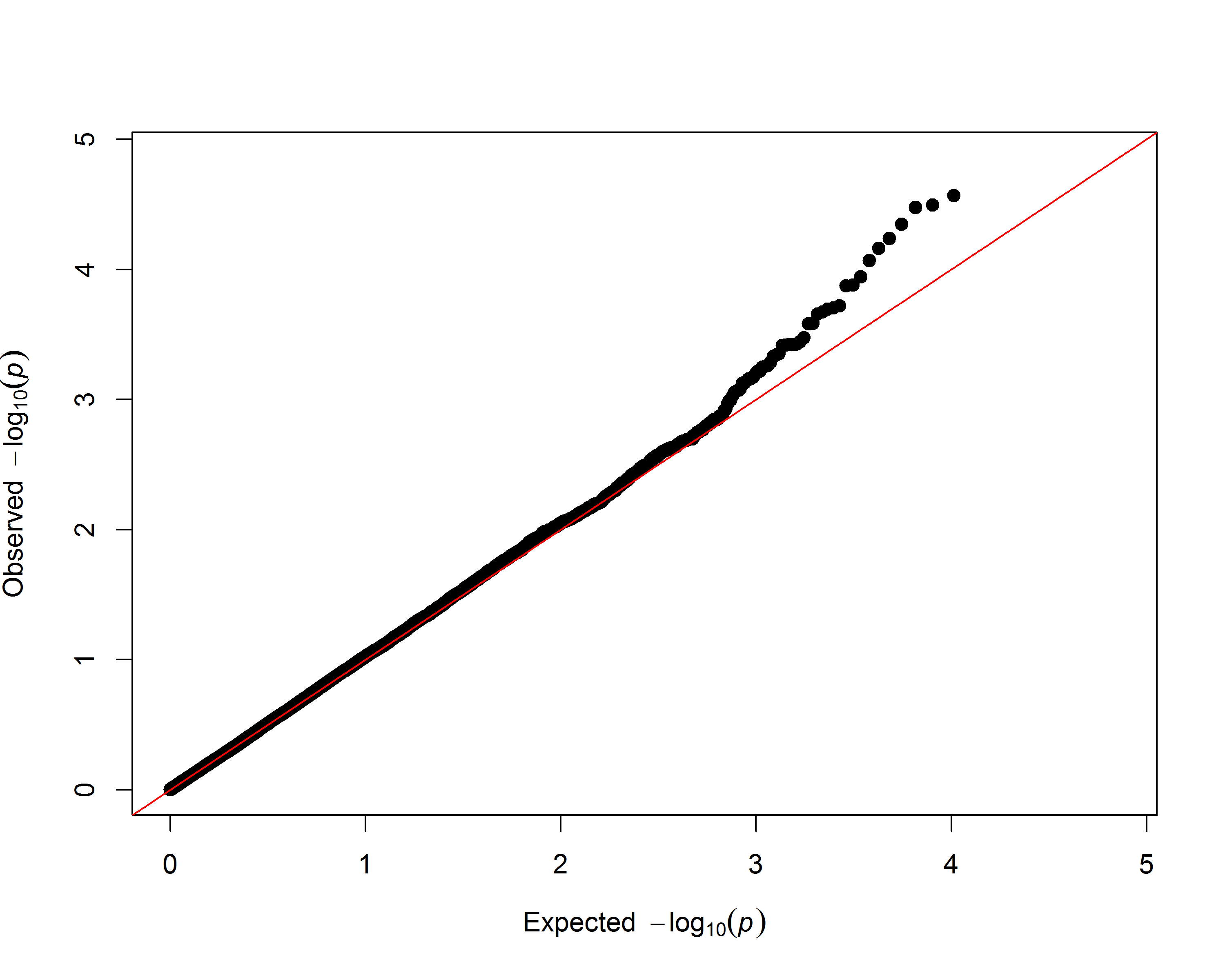

Supplement: Figure S1 — Quantile-quantile plot for apnea hypopnea index in African-Americans. This figure plots expected versus observed p-values from the association analyses of all SNPs against log(apnea hypopnea index+1) in African-Americans. The plotted observed p-values are after accounting for genomic control. (TIF) [file pone.0048836.s001.tif]

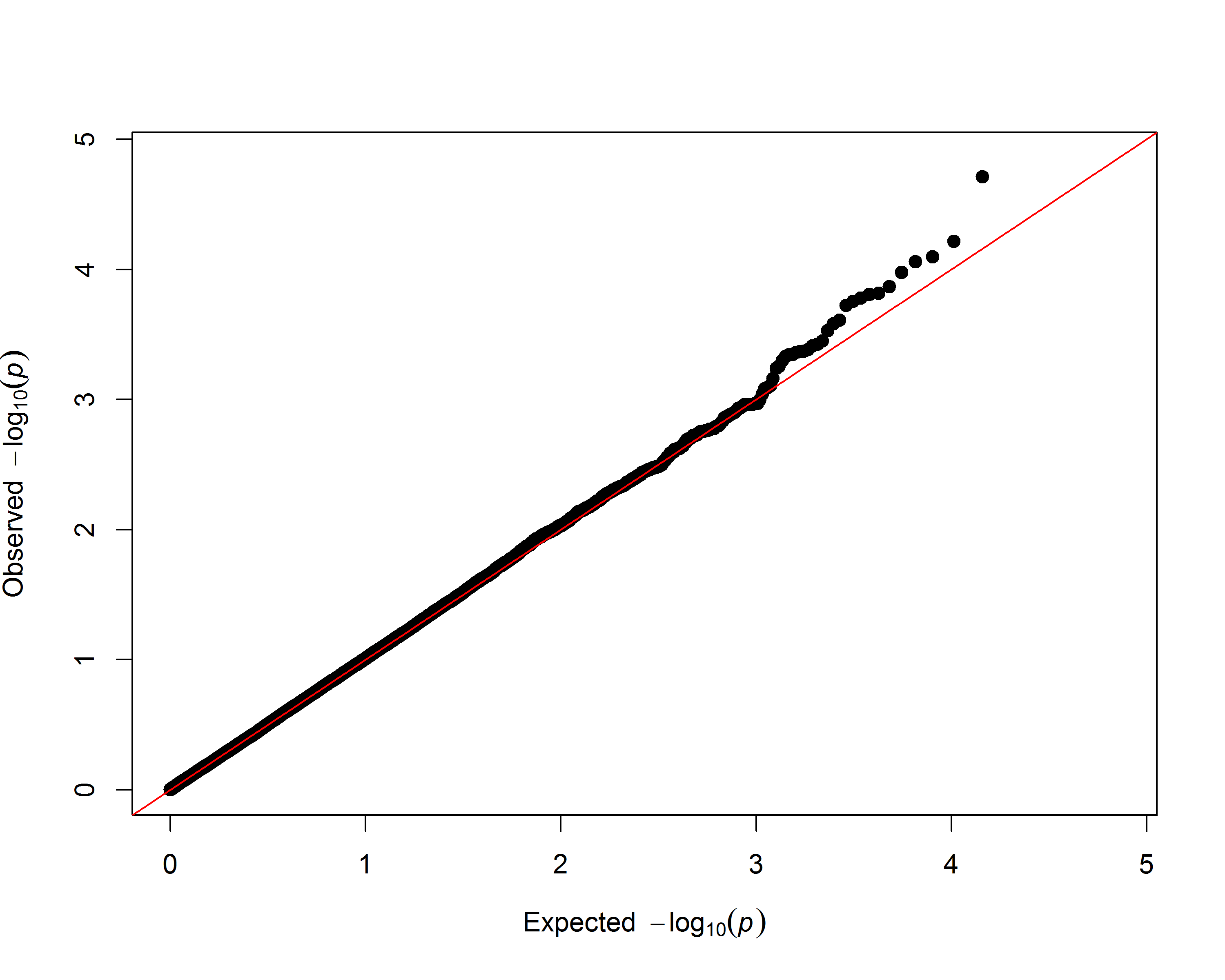

Supplement: Figure S2 — Quantile-quantile plot for obstructive sleep apnea in African-Americans. This figure plots expected versus observed p-values from the association analyses of all SNPs against an apnea hypopnea index of 15 or greater in African-Americans. The plotted observed p-values are after accounting for genomic control. (TIF) [file pone.0048836.s002.tif]

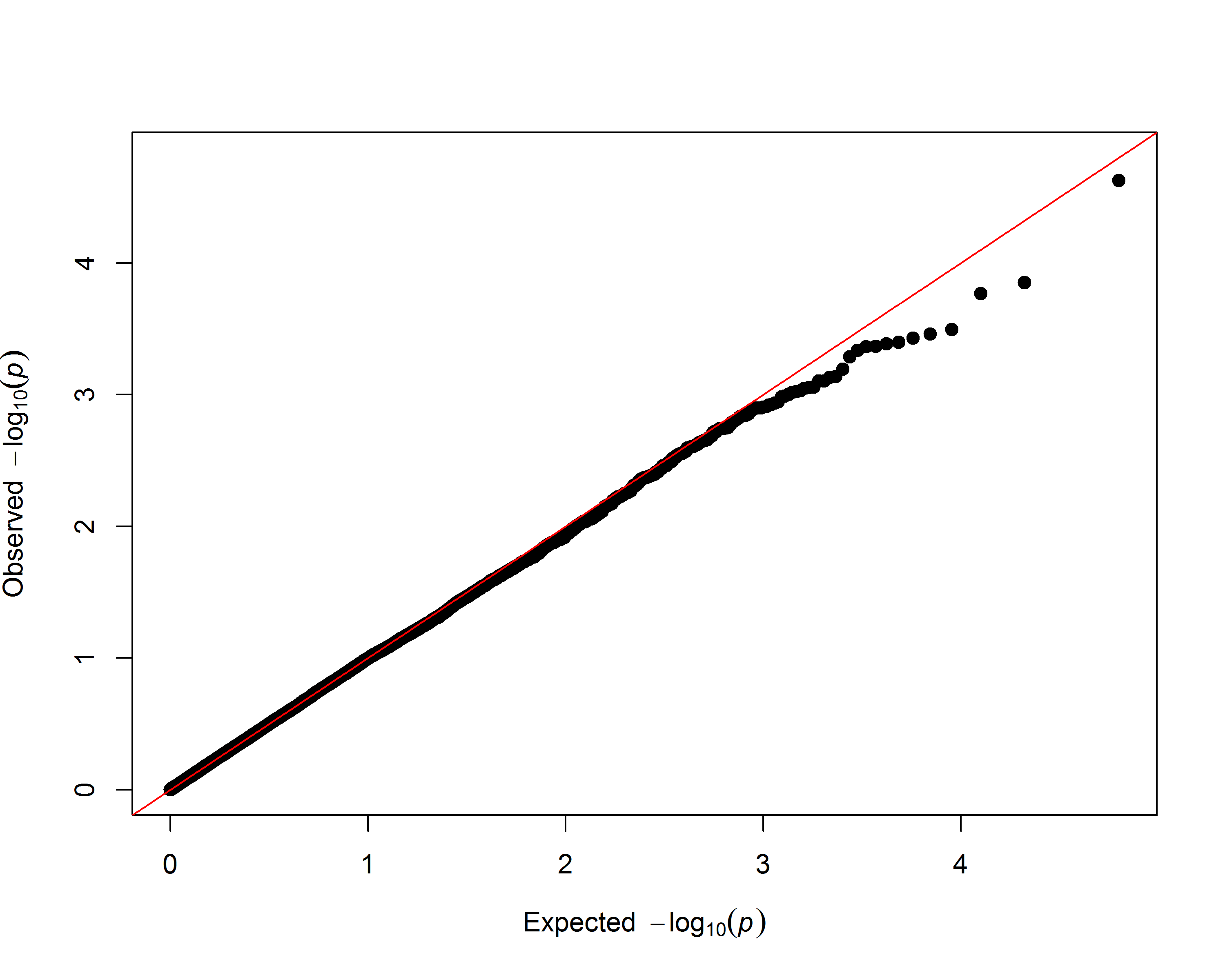

Supplement: Figure S3 — Quantile-quantile plot for apnea hypopnea index in European ancestry individuals. This figure plots expected versus observed p-values from the association analyses of all SNPs against log(apnea hypopnea index+1) in those of European ancestry. The plotted observed p-values are those from the meta-analysis after accounting for genomic control. (TIF) [file pone.0048836.s003.tif]

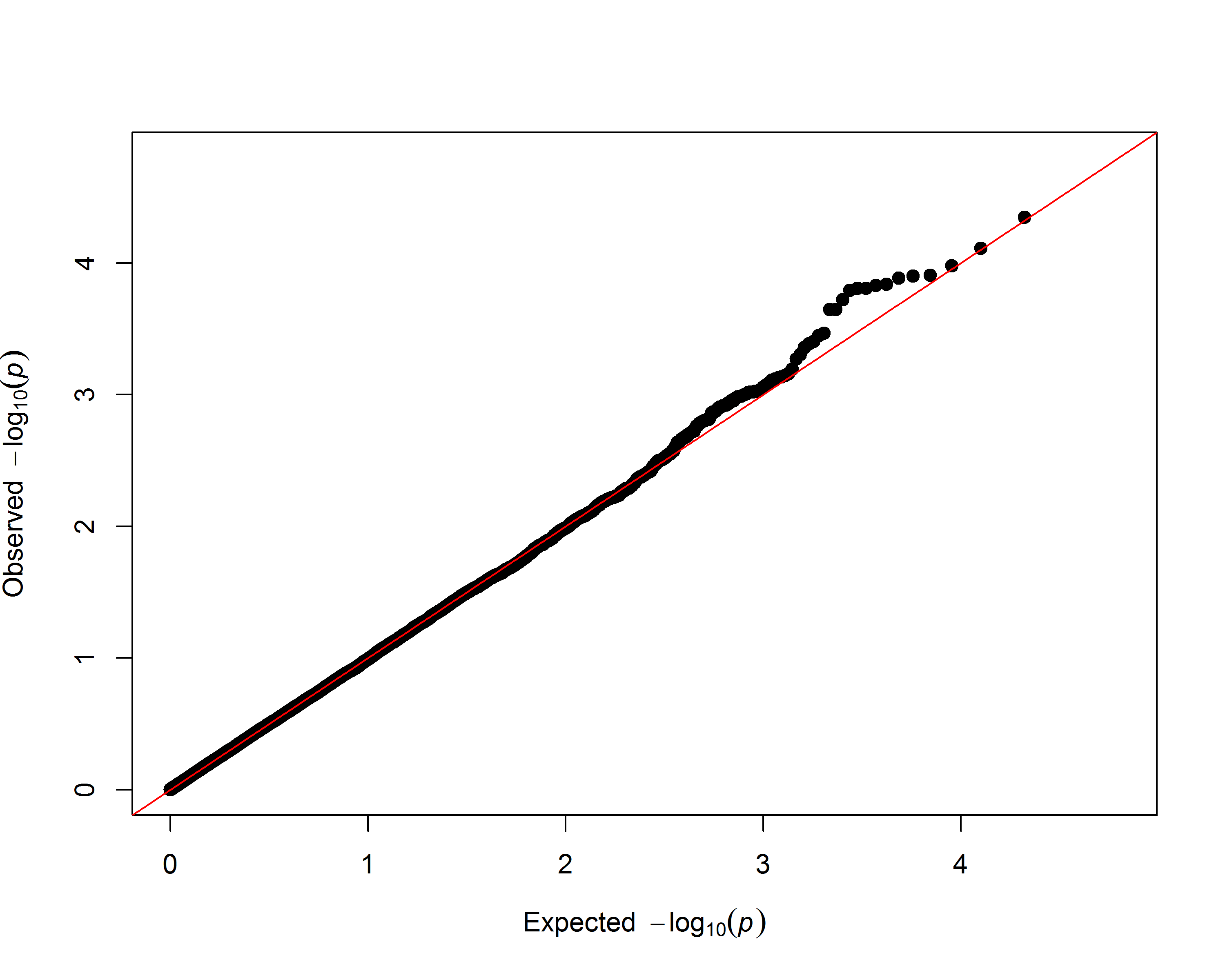

Supplement: Figure S4 — Quantile-quantile plot for obstructive sleep apnea in European ancestry individuals. This figure plots expected versus observed p-values from the association analyses of all SNPs against an apnea hypopnea index of 15 or greater in those of European ancestry. The plotted observed p-values are those from the meta-analysis after accounting for genomic control. (TIF) [file pone.0048836.s004.tif]
